# An examination of large-scale electronic health records implementation in Primary Healthcare Centers in Saudi Arabia: a qualitative study

**DOI:** 10.3389/fpubh.2023.1121327

**Published:** 2023-08-09

**Authors:** Haitham A. Alzghaibi

**Affiliations:** Department of Health Informatics, College of Public Health and Health Informatics, Qassim University, Albukayriah, Saudi Arabia

**Keywords:** electronic health records, large-scale IT project, Primary Healthcare Centers, IT project management, healthcare delivery

## Abstract

**Introduction:**

Digital transformation has become a buzzword in almost every industry in the twenti first century. Healthcare is not an exception. In the healthcare industry, digital transformation includes the utilization of electronic health records (EHRs), telemedicine, health information exchange, mobile health, and other interactive platforms. The importance of digital transformation in healthcare cannot be overemphasized as it has proven to be critical in improving patient outcomes, making healthcare delivery more efficient, and reducing costs. The positive impact of electronic health records was noticed almost immediately in the field of primary healthcare. It has been suggested that implementing electronic health records will enhance the accessibility and the process of distribution of health records between authorized users. As part of Saudi vision 2030, all healthcare organizations in Saudi Arabia are going to shift to digital transformation.

**Methods:**

This study follows a qualitative, semi-structure, face-to-face interview approach. The collected data were analyzed using NVivo V10 software. Inductive thematic analysis approach was used to analyse the collected data from the interviews.

**Result:**

Seventeen project team members, from different positions and backgrounds were purposively chosen to be interviewed. Three main themes and 38 codes were generated from the analysis of transcripts. The informants describe the implementation of electronic health records in the PHCs based on two different experiences. The participants reported that a previous attempt failed due to inappropriate infrastructure, lack of technical support, and low level of user acceptance. Therefore, the policymakers adopted several steps to increase the level of success and avoid failure causes. They initially established well-defined requests for proposals followed by continuous commendation among the project team and conducted a consultation on multiple levels (country level; organizational level and individual level).

**Conclusion:**

This study concluded that the main causes that lead to the failure of the large-scale project were lack of connectivity, lack of technical support, and staff changes, particularly those who occupied high-level positions in the Saudi ministry of Health. The success rate of EHRs implementation can be directly impacted by the size of the project. Large-scale projects are complicated and may be subject to numerous challenges compared with small projects. Significant factors such as training, support, legal issues, and organizational workflow and redesign were a concern of the project team during the pre-implementation phase. In addition, other factors related to technology and end-users were included in the EHRs implementation plan.

## 1. Introduction

The introduction of Electronic Health Record System (EHRs) in Saudi healthcare organizations is considered to be one of the highest priorities of policymakers (1). However, there is currently a dearth of available literature, particularly in the field of EHRs implementation assessment in SA. Each country has its own unique and individual culture and systems, and if EHRs implementation is to be successful in the long term, it must conform to the distinctive customs and traditions of each country (2). Despite having some characteristics of a traditional organization, the healthcare sector is different from other sectors, mostly due to the complexity and social hierarchical structures both within and between institutions (3, 4). Therefore, research carried out in other sectors cannot always be applied directly in the healthcare sector. A thorough examination of the research on EHRs implementation in the healthcare context and from the perspective of the organization is therefore necessary. Despite the remarkable growth in the volume of published research determining and defining EHRs implementation in the past few decades (5, 6), the published research has focused on secondary care organizations or small-scale projects (6). This proposed research is important due to the limited research that has focused on large-scale projects, in particular those related to implementation in Primary Healthcare Centers (PHCs).

Since the 1980s, HI has shifted toward the digital transformation of hospital administration and financial aspects (7, 8). This process of digital transformation is straightforward, simple, automated, and also economical. With the advancement and enhancement of clinical systems, secondary EHRs were also being computerized. The typical secondary EHRs becoming computerized are laboratories, radiology departments, and pharmacy departments (7). According to Gagnon and Desmartis (9), the first use of EHRs in PHCs was in the 1990s.

The positive impact of EHRs was noticed almost immediately in the field of primary healthcare (10). It has been suggested that implementing EHRs will enhance the accessibility and the process of distribution of health records among authorized users (11–15). In addition, EHRs can improve the economic and administrative abilities of all healthcare institutions and, also, directly influence the quality of care provided to patients (16–20). Despite the positive impact of EHRs, the implementation process may be subject to several challenges such as EHRs interoperability. EHRs interoperability has been highlighted as an influential factor in its implementation (6, 15, 18, 21–25). The basis for systems interoperability lies in a common understanding of the data codes and concepts among EHRs (25–27). Unsuccessful interoperability may occur due to misconnection or discoordination between providers of different healthcare organizations, as different organizations utilize different types of standards (25, 27). Arvanitis (26) divided systems interoperability in healthcare into two dimensions, titled “*syntactic interoperability”* and “*semantic interoperability”*. Semantic interoperability is a core factor in large-scale projects (28).

The Saudi government has allocated four billion Saudi Riyals (the equivalent of $1066 million) to the establishment of the National Electronic Health Record (NEHR) in the future and for the e-health strategy to be accomplished (29). Currently, more than 70 projects have been identified to achieve this e-health vision (30). The MoH of SA plans to implement EHRs in all PHCs following the previous failure of other projects at a cost of billions of Saudi Riyals (31).

Whilst studies in the field of EHRs have been conducted in many countries, there is no existing research on the implementation of large-scale EHRs at PHCs in SA. Moreover, as mentioned earlier in this chapter, each country has its unique system. Hence, SA may face a unique challenge and also benefits from unique facilitators. Each implementation project has its challenges and facilitators. Therefore, the level of success or failure may vary based on the project scale, organization size, and place of implementation (32, 33). Causes of failure or success may not be applicable for all types of implementation projects due to this variance. The challenges that hinder the success of EHRs implementations encouraged the author to investigate and explore the implementation of large-scale projects, which can be more complicated and may face more challenges. It would be useful to examine the underlying causes and issues that influence the success of EHRs implementation. Therefore, this study aims to explore the large-scale implementation of EHRs in PHCs in SA.

## 2. Methodology

The decision to conduct semi-structured interviews was made based on the ability of interviews to provide in-depth, rich, and detailed information which cannot be obtained via questionnaire-based approaches. Therefore, to achieve the study's aim, further qualitative research was conducted via semi-structured interviews (34).

### 2.1. Study sampling and population

Policymakers and other project team members were identified as the most appropriate individuals to provide an in-depth description of EHRs implementation. The study population comprises all project team members directly or indirectly involved in implementing a large-scale EHRs project in Saudi PHCs. These consisted, for example, of heads of relevant departments [Information Technology (IT) and PHC departments], senior managers, IT engineers, and technicians. This potential population of participants within the Saudi MoH has varying backgrounds and experience, departments, occupations, and genders. The target sample was therefore all project team members (*n* = 53).

Of those invited, only 17 agreed to take part in an interview. The purposive sampling technique was selected, as key informants were required to ensure they had a wide knowledge of the project. For instance, the majority of the items in the interview guide required the participants to provide details and in-depth information about the EHRs and the barriers and facilitators they faced during the implementation process. In this study, bias in purposive sampling has been reduced by conducting a sufficient number of interviews until no new information emerged.

### 2.2. Data collection via semi-structured interviews

In this study, data were collected using semi-structured interviews. The type of questions used were open-ended, to allow the participant the flexibility to describe their views and opinions. Semi-structured interviews helped expand on the questions following unexpected or interesting responses. Semi-structured interviews can also gather a wider variety of detailed data. Therefore, the main purpose of conducting semi-structured interviews was to gain a comprehensive understanding and explanation of the full process of EHRs implementation in PHCs.

This included a detailed plan of how certain procedures were to be implemented, for example, the methods used to provide training and support. In addition, semi-structured interviews were selected because they are more flexible and allow the author to discuss additional issues that are not covered in the interview guide. This allowed the author to obtain insight into the perceptions and attitudes of the project team.

### 2.3. Data collection process

A total of 17 one-to-one interviews took place with the selected participants. The interviews were conducted over a 4-month period. Two-hour slots have been scheduled for each interview; however, no time limits were applied with regards to how long the interview could last. Participants were informed that they could withdraw from the interview at any time. In addition, to make the interviews as convenient as possible for the interviewees, the author traveled to the participants to interview them.

The author arrived at least 30 min before the interview time to make sure that the interview location was suitable. With the exception of one interview, all were conducted at the same location. In addition, all the participants were presented with the same questions, in accordance with the guidelines for conducting interviews. Moreover, field notes were taken during the interview to avoid interruption when new questions emerged during the interview. This allowed the interviewer to generate more questions to overcome the omissions and also to clarify any comments made. This also allowed flexibility in the interview and helped the interviewer identify any comments that could lead to new questions and fields of research. In addition, some field notes were taken during the interview to capture body language and participants' reactions that could not otherwise be detected in the audio recording.

All interviews were digitally recorded using an iPad and an iPhone. The memory available on both devices was capable of recording audio for up to 50 h. Two devices were used to ensure that one source was always available if the other device was lost or damaged during the interview. Each device was kept opposite to the other to record the audio accurately. The voice recording files were uploaded to the author's laptop and checked to see if there were any obvious issues during the recording. All voice files were clear, which assisted in the transcription of the interviews.

### 2.4. Bias in qualitative research

Semi-structured and unstructured interviews may face bias issues (35) because, in qualitative research, the researcher is typically considered to be the instrument of data collection (35). Bias in interviews can occur as a result of two main issues: researcher performance and the data collection instruments (interview guide) (35).

#### 2.4.1. Role of the researchers

The author took care to minimize any potential for bias. The author tried to make the interviews as friendly as possible. As mentioned earlier, the author spent sufficient time in the field to be familiar with the context and also become familiar with the participants, in particular those who participated in the semi-structured interviews. In addition, all participants were informed of the ethical considerations to ensure their privacy and confidentiality. They all received a copy of the ethical approval and consent form. The author informed the participants that their data would not be used for any purposes other than this research and that no one else would have access to the transcripts. In addition, they were told that the data would not be used for evaluation or audit purposes and would not be provided to anyone in their organization. The author also stated that the author would send them a copy of the transcript for validity purposes before they were used. Finally, the author explained to them the value of their responses and how they could contribute to the research.

Participants were allowed to choose whether to be interviewed in English or Arabic to avoid any potential for misunderstanding. The author also tried to ensure that the interview location was quiet and the interview could not be heard by others. This was to ensure that the participants could answer questions without fear of being overheard. The author took into account that the role of the interviewer was to listen more than speak during the interview. The author, therefore, allowed the participants to talk without any interruptions and avoided making comments or asking other questions. The author also avoided showing any facial expressions or body language that could have influenced the participants' answers or changed their opinions. The author took care not to provide any suggestions or alternative answers to the participants, even if they did not fully answer the question. In addition, the author ensured that sufficient time was given to the participants to allow them to adequately answer the question, express their point of view, or describe the process. The author avoided confirming participant opinions, even if these opinions were in agreement with the author's hypotheses. The author, therefore, remained neutral at all times.

#### 2.4.2. Interview guide-related bias

Regarding the bias related to the interview guide, the author took into consideration when asking questions that they would not lead to a specific answer. In addition, there are no sensitive questions or questions that were likely to result in a particular socially desirable response.

### 2.5. Qualitative data analysis of semi-structured interviews

The purpose of qualitative data analysis is to “make sense of the collected data” (36). The researcher can utilize computer software to collect and analyse qualitative data (35). NVivo V10 (QSR International, Denver, CO, United States) software was used to thematically analyse the qualitative data. NVivo software helps to manage rich text by categorizing it and organizing it rather than analyzing the text, as other quantitative programs do.

The qualitative data collected in the semi-structured interviews were analyzed using thematic analysis. Thematic analysis is considered to be flexible and accessible and assists the researcher in providing rich and detailed descriptions of the data (37). Thematic analysis is thought to be the foundation of qualitative data analysis (37). According to Braun and Clarke (37), thematic analysis helps to capture significant information that assists in describing the research question.

Thematic analysis was selected because it was considered to be flexible and not restricted to a specific framework or theory. In addition, the decision was made to perform thematic analysis to identify patterns in extremely rich information from different perspectives (34). Thematic analysis was found to be useful to identify patterns and themes that represented implementation procedures.

### 2.6. Transcription and translation checking

Once all interviews were conducted and transcribed, the data that would identify any individuals were removed and then the transcribed data were compared against the original audio file to ensure accuracy. Thereafter, Arabic transcripts were sent to an official translation agent to ensure the translated data were correct. Thereafter, all transcripts were sent to another independent researcher who has experience in thematic analysis for other observations. The inter-rater conducted the above six stages of thematic analysis independently. Although there was about 80% agreement regarding most of the themes and codes generated, there was disagreement about some of the codes and their quotes. English is the author's second language and also that of the independent inter-rater. As a result, at times, different terms were used for similar codes.

## 3. Result

This study was designed to explore the implementation of EHRs in Saudi PHCs from a project team perspective. The readiness assessment will take into consideration a description of the implementation plan and other factors influencing EHRs implementation in the pre-implementation phase. A thematic analysis was used to analyse the obtained data. The selection of the quotations was based on a line-by-line reading of the transcripts, and all quotations included in this study have been selected for their appropriateness to the study's aims. The included quotations are directly related to the above-mentioned aims.

The participants occupied five different positions (see Table 1): General Manager (*n* = 3), Head of Department (*n* = 3), Deputy Head of Department (*n* = 3), Software Developer (*n* = 4), and DA (*n* = 4).

**Table 1:** Participants position and abbreviation.

**No**.	**Participant positions**	**Abbreviation**	**Number of participants**
1.	Software developer	SD	4
2.	Data analyst	DA	4
3.	General manager	GM	3
4.	Head of department	HD	3
5.	Deputy head of department	DHD	3
		Total	17

**Figure 1:**
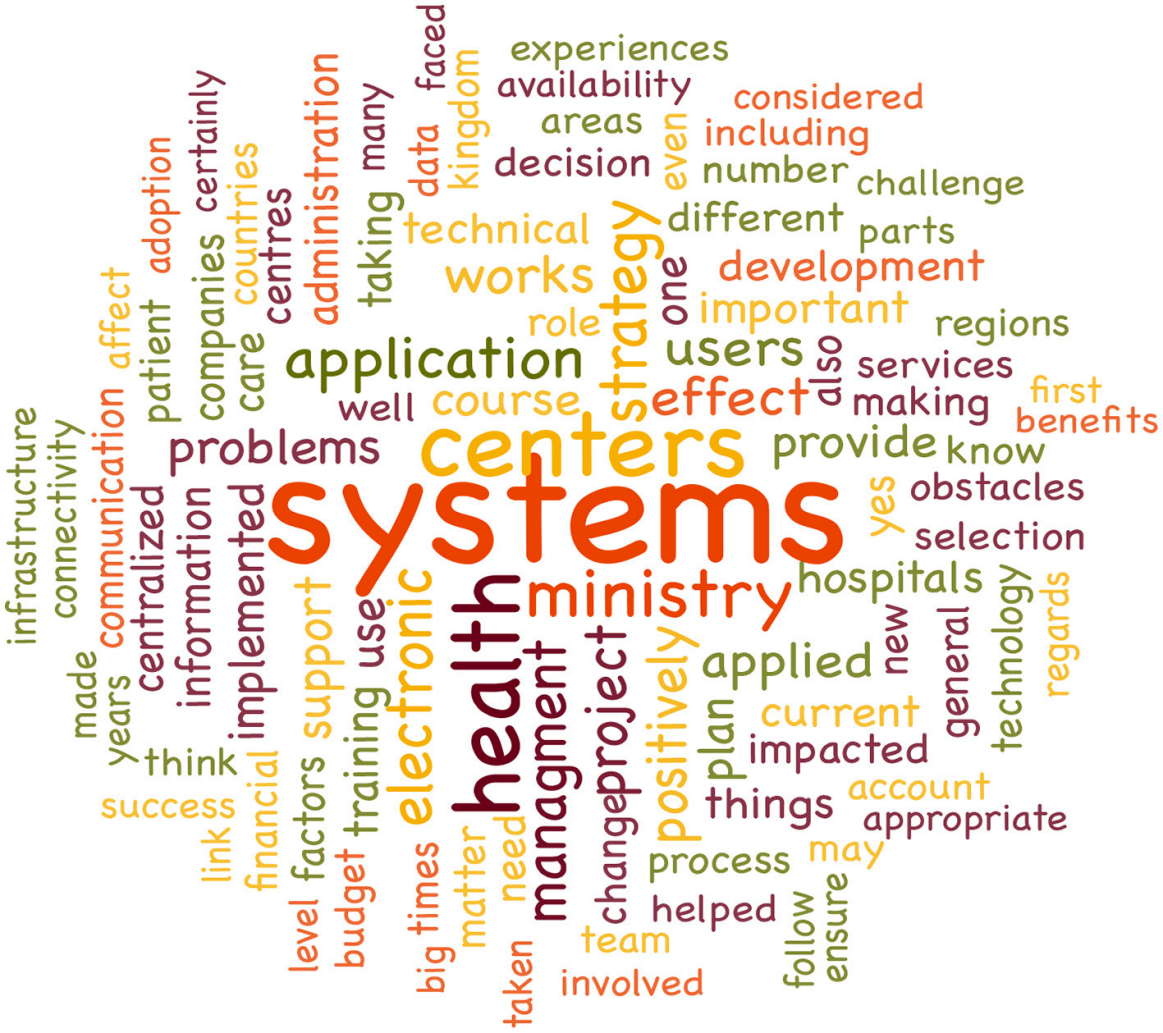
The most frequent words in the interviews.

Figure 1 shows the most frequent words and terms reported during the interviews. “Systems”, “health”, and “centers” were the most frequently mentioned. The words included in Figure 1 are the most frequent, and other words in the transcripts are not available in the figure.

As seen in Table 2, 38 key codes emerged from the analysis of the transcripts. Although the number of codes that emerged from the transcripts was greater than included in the table below, Table 2, includes the key codes only. All codes and sub-codes will be presented in the following sections. Three main themes were phrased according to all codes that emerged from the transcripts:

- Theme one: Challenges of the previously implemented EHRs.
- Theme two: Future plan to implement large-scale EHRs in PHCs.
- Theme three: Procedures adopted in the pre-implementation phase.

**Table 2:** Key codes and sub-codes.

**No**.	**Key codes**	**Sub codes**
1.	Restructuring and workflow redesign of PHCs	
2.	Readiness assessments to new technology	
3.	Conduct the consultations	
4.	Project team selection	
5.	Consultation	
6.	Project team communication	• Communications are made via committee • Communications are made via workshops • Communications are made via meetings • Communications are made via media
7.	Awareness campaigns to EHRS users	
8.	The role of management and leadership on the implementation process	
9.	Training provision	• Training delivery methods • Training provision as a condition of the contract with vendors • Training provision timeline
10.	Technical support provision	Provision of technical support as a condition of the contract with vendors
11.	Security, privacy and confidentiality assurance mechanisms	•Data protection laws to protect patient data • Monitoring and auditing by PHC directors • Limited access privileges • Selecting a secure system
12.	End-user involvement mechanisms	
13.	User acceptance to new EHRS implementation	
14.	User resistance to new EHRS implementation	
15.	EHRS user requirements	
16.	Software selection criteria	• EHRS Usability • EHRS Efficiency • EHRSs Interoperability • Local or international system • Request for Proposal (RFP)
17.	The level of infrastructure	
18.	Financial support	
19.	Characteristics of the PHCs and its impact on the EHRS implementation	
20.	Perceived usefulness of the EHRS	
21.	Changing Key people and its impact on the EHRS implementation	
22.	Lack of experts in EHRS implementation projects	
23.	Number of PHCs	
24.	EHRS implementation timescale	
25.	Vendors selection and contract	
26.	Lack of connectivity	
27.	Geographical challenges and its impact on the EHRS implementation	
28.	Piloting the EHRS	
29.	Co-operation with Telecommunications Companies (TCs)	
30.	Divide the country into regions	
31.	Conducting Studies and research	
32.	Technology developments	
33.	Continues evaluation	
34.	User awareness and readiness to new EHRS	
35.	Evaluation of the previously implemented EHRS	
36.	Perceived usefulness of the previously implemented EHRS	
37.	Technical support issues related to the implemented EHRS	
38.	Training issues related to the implemented EHRS	
39.	User awareness about the previously implemented EHRS	
40.	User resistance to the previously implemented EHRS	
41.	Connectivity issues as failure causes	
42.	The efficiency of the previously implemented EHRS	
43.	Usability issues of the implemented EHRS	

### 3.1. Theme one: challenges of previously implemented EHRs

The Saudi MoH has previously implemented EHRs in approximately 150 PHCs.

“*I think it is 150 PHCs.” (GM 1)*

“*It was implemented in 150 PHCs 10 years ago.”* (GM 2)

However, this project failed due to several factors such as changes at the administrative level, particularly at the level of ministers and senior managers.

“*Unfortunately, with administrative changes and the change of the former minister, work on the current EHRs was terminated.”* (HD 3)

#### 3.1.1. Evaluation of the previously implemented EHRs

The Saudi MoH IT department evaluated the implemented EHRs regularly.

“*We were doing an assessment of the system on a regular basis.”* (GM2)

“*It was being evaluated from time to time.”* (HD2)

“*The EHRs was evaluated by colleagues in the IT department.”* (HD 1)

The evaluation process is performed using two different methods. The first method involves sending a team from the MoH to test the system on-site, while the second method involves holding meetings with EHRs end-users and asking them to fill in a questionnaire to ascertain their views on the system.

“*One of the assessment methods was sending teams to the PHCs to test the EHRs and stand on its problems.”* (GM2)

“*I was holding a meeting with the end-users themselves and asked them to fill in a questionnaire.”* (DHD1)

However, others argued that no evaluation of the implemented EHRs had been performed.

“*No evaluation has been done.”* (DA 2)

“*No assessment of the implemented EHRs has been done.”* (HD3)

#### 3.1.2. Perceived usefulness of the previously implemented EHRs

Although the previously implemented EHRs have been considered a failure, it does have a positive impact on PHCs. Thus, the performance of PHCs has changed for the better, which has resulted in a major improvement in services provided to patients.

“*Undoubtedly the EHRs changed the PHCs for better.”* (HD 2)

“*The EHRs changed positively because it raised the level of performance and efficiency.”* (DHD 1)

Moreover, the performance of the staff in these PHCs has been positively affected. In addition, experience with using EHRs has motivated those users to implement new EHRs after the termination of the previous system. The former EHRs assisted in making users more aware of modern technology and adapting to the needs of the future.

“*The system made changes to user performance for the better.”* (DA 1)

“*It created a desire among users to implement the new EHRs, and they are motivated to use the new system.”* (DHD 3)

#### 3.1.3. Technical support issues related to the implemented EHRs

Technical support was considered one of the main challenges that the previous EHRs project faced. The Saudi MoH received numerous complaints from EHRs end-users due to the lack of technical support.

“*We faced a problem with maintenance, many users complained of lack of maintenance of the system.”* (GM2)

Moreover, the lack of technical support resulted in disappointment for many EHRs end-users and led to them being resistant to the system.

“*Technical support was a source of concern for users. Among the problems I remember, therefore, we faced some opposition from the end-users, they were not willing to use the system due to lack of technical support.”* (DHD 1)

One of the general managers argued that the lack of technical support was one of the causes that led to the failure of the previous EHRs.

“*The EHRs failed because it was without technical support.”* (GM 3)

#### 3.1.4. Training issues related to the implemented EHRs

When examining the provision of training to end-users of the implemented EHRs, some participants agreed that all EHRs end-users have received adequate training since the first day of implementation. For example:

“*There was a very adequate training course.”* (HD3)

“*Staff were trained from the first day the EHRs was implemented.”* (SD1)

Moreover, the training given was accredited by the Saudi MoH. The training sessions took place at different times and at different locations. It was noted that the training courses were carried out both inside and outside working hours as well as inside and outside PHCs. In addition, continuous training was provided to the PHC staff.

“*Training courses are held continuously, as needed; and courses are provided inside and outside the centers.”* (DHD 1)

“*We had continuous training courses which were held outside work times and within work times.”* (GM 2)

However, others argued that not enough training courses were provided for the EHRs end-users.

“*There are not sufficient courses at the present time; they are few.”* (HD 1)

On the other hand, others stated that there are no training courses; and training has been suspended for the former EHRs.

“*At present, there is no training.”* (DA 1)

“*Currently, the system is disrupted, and training sessions have stopped as well.”* (SD 1)

#### 3.1.5. User awareness about the previously implemented EHRs

EHRs end-users were fully aware of the EHRs implementation project. At the beginning of the project, there was an awareness campaign conducted by distributing brochures and sending announcements and circulars to the targeted PHCs. These contained information on the benefits of the system and its role in facilitating their tasks and improving the healthcare provided. In addition, the brochures contained a simple explanation of how to use the system.

“*We made announcements and circulars stating that we will develop a system to be implemented in the PHCs. It also stated that the role of the EHRs will assist the users in providing high quality services to patients from the time they enter the PHC until they are released. We have also distributed posters and brochures.”* (SD 1)

#### 3.1.6. User resistance to the previously implemented EHRs

User acceptance varies from one PHC to another.

“*The level of system acceptance varies from one PHC to another; some centers were enthusiastic and willing to apply it, whereas other centers have no desire.”* (SD 1)

Some of the interviewees stated that the Saudi MoH had faced difficulties associated with the end-users themselves, as some of them were unwilling to use the EHRs. This is a major obstacle that may lead to implementation failure.

“*We face some resistance from end-users.”* (DA 2)

“*We encountered some resistance.” (GM 2)*

“*Some of the staff don't want to use the EHRs.”* (SD 1)

There are several reasons for the reluctance to use EHRs. According to SD1, one of these is computer illiteracy; it was found that users who do not have experience in using computers are unwilling to use the system. Another reason is that the PHC directors themselves did not seem willing to provide any support.

“*We face some problems from users who have no computer experience - they do not want the system.”* (SD 1)

“*We don't have appropriate support from the PHCs that we implemented the system in; especially the centres' directors. We didn't get the expected support from them.”* (SD1)

HD2 argued that the most likely reason for the end-user's resistance and unwillingness to use the system was the lack of full familiarity with EHRs and a lack of awareness of the purpose of their implementation in PHCs. Therefore, this participant provided a solution to overcome this issue, stating, “*This problem is solved through training”*.

“*In some cases, the lack of desire to use the system was caused by lack of understanding of how to use the EHRs.”* (HD2)

Another reason was mentioned by DA 2, who perceived that older users are often more resistant. This is not confined to the use of EHRs, but also to the use of computers in the workplace in general.

“*Many users, especially the older users, do not want to deal with computers at work.”* (DA 2)

It has been illustrated that end-users may not be willing to use EHRs in their workplace and may refuse the change. They believe that the new system may negatively affect their work routine and may even lead to them losing their jobs.

“*It is very natural to find a person who doesn't want to change; most of the employees do not want to change from one system to another EHRs; they think this change may threaten their job security”* (GM2)

Finally, DA 1 revealed that some end-users still prefer to use a paper-based system instead of using an electronic-based system.

“*Some users prefer to stay on the paper system.”* (DA 1)

#### 3.1.7. Connectivity issues as failure causes

Connectivity is another challenge to the implementation of EHRs in PHCs. In this context, linking PHCs is almost impossible, due to lack of internet connection.

“*The connection of PHCs with each other is difficult, it's nearly impossible.”* (GM 1)

“*The main problem was the impossibility of connecting the EHRs in the PHCs with the internet.”* (DA 1)

Moreover, connectivity issues are directly associated with the failure of the previous EHRs implementation project.

“*Connectivity between the PHCs is considered a big challenge which led to failure of the project.”* (DA 1)

However, “*A few PHCs have successfully been linked to the Internet.”* (HD2)

#### 3.1.8. The efficiency of the previously implemented EHRs

When examining the previous EHRs, inefficiency was found to be another reason for implementation failure. In addition, one of the heads of the departments described the previously implemented EHRs as “a *modest system”*. Moreover, decreased efficiency of the implemented EHRs was considered to be due to a lack of comprehensiveness, where the required functionality to provide a proper healthcare service to the patients was not being integrated. Therefore, the previous system was lacking many of the commands and features compatible with the functions of PHCs.

“*The implemented EHRs is a modest system and not one of high efficiency.”* (HD 3)

“*They implemented EHRs, but they were not comprehensive and did not have all the required characteristics and functions, such as a CDSS. The coding in the system differs from the user's coding system in the Ministry, and this is one of the defects which curtailed the advantage of the current EHRs.”* (GM 1)

In addition, the implemented EHRs did not meet the expectations of the end-users.

“*As far as I am concerned, the previous EHRs does not meet users' expectations and it was not fully efficient.”* (DHD 2)

“*I believe that the current EHRs has not come up to the expectations of the users.”* (HD 2)

However, the software developer argued that the implemented EHRs were efficient enough to meet the end-user's expectations.

“*The EHRs meets the requirements of the PHCs more than you think; the proof is that there are still some PHCs using the EHRs.”* (SD1)

#### 3.1.9. Usability issues of the implemented EHRs

Some participants stated that the end-users of the EHRs found the system easy to use. For instance, DA 2 stated, “*All users agreed that the system is very easy to use”* (DA 2), and GM1 stated, “*The EHRs is honestly easy to use”*.

However, according to the software developer, some doctors expressed dismay at the number of screens, particularly during movement between commands, and time was wasted as a result.

“*The flow of many screens has upset some doctors. They do not want to move through many screens and from one screen to another - that takes a lot of time.”* (SD1)

### 3.2. Theme two: plan for EHRs implementation in PHCs

This theme describes in detail the process to formulate a plan to implement new EHRs in PHCs. The participants highlighted the main elements of the plan as well as the process. Initially, everything is still on paper, and no practical steps have been taken. The Saudi MoH has developed an elaborate scheme for the implementation based on the principle of priority, whereby projects are implemented in certain PHCs according to preliminary needs.

“*All that has been done before is a theoretical thing on paper; there has been no action taken or a trial for certain systems.”* (GM 2)

The essential stage in the planning phase was to conduct several consultations at various levels.

#### 3.2.1. Consultations during the formulation of the EHRs implementation plan

In this section, the participants describe the need for consultation with this type of project and how such consultations are conducted. The Saudi MoH has carried out several different consultations to enhance the implementation plan, conducted on three different levels: country, organizational, and individual.

• Consultations at the country level:
° Canada, Australia, Turkey, Norway, Jordan, Denmark, United Kingdom, United States, South Korea, and Singapore.
• Consultations at organizational level:
° Health Info Way and IBM.
• Consultations at individual level:
° Reviewers, auditors, and consultants from SA and some developed countries such as UK and Australia.

##### 3.2.1.1. Consultations made at the country level

Although the Saudi MoH has conducted consultations in different countries, the nature of the Saudi PHCs business process and workflow is different from that of other countries, as this participant's comment highlights:

“*We benchmark with the Canadian experience, and we also had a benchmark with some countries like Australia, Turkey and Denmark, which have big initiatives.”* (GM 1)

“*In addition to America, we have a number of trips and visits to a group of countries in Europe (Norway, Spain, Germany, Italy), North America (Canada) and South Korea. We have access to all the systems they already have, and we have benefitted from their experiences in this area.”* (HD 1)

“*Moreover, we benefited from the experiences of other countries like Norway and Australia.”* (DHD 3)

“*The Ministry benefited from the South Korean experience as well as the Jordan experience.”* (DA 1)

“*Most of the benefits that we have are from the United Kingdom (UK) experience; the UK experience is a very rich experience with a lot of difficulties and failures, so it was very rich, and we learned from it.”* (GM 1)

The consultation-based reviews varied from country to country depending on several criteria. One of the criteria considered was the resemblance of the Saudi healthcare system to that of other countries. The healthcare system of some countries, such as Canada, is considered closest to the Saudi healthcare system. Thus, there is great co-operation between health institutions in SA and Canada in this context. Subsequently, there is Australia, where the healthcare system is the second most similar to that of Saudi Arabia. Singapore occupies third place in terms of similarities with the SA healthcare system. Therefore, these countries are the most involved in the consultation process based on the statements of the participants; for example:

“*The countries most involved are Australia and Singapore.”* (DA 3)

“*…especially since Canada is the nearest country to us in terms of its healthcare system and sharing some of the challenges such as cities that are a great distance from each other, so there was co-operation between us directly and continuously.”* (HD 1)

As stated by a head of the department, “*It is not logical to use other countries' strategies and apply them to SA, or even take some aspects”* (HD 1). Therefore, the purpose of the consultations was to take advantage of the positive experiences of different countries, regardless of the differences in healthcare provision and organization. The countries that were successful in implementing their strategies are the UK, Canada, and America; as indicated in the following comments:

“*Definite strategy in the beginning was to bring distinctive resources of the strategies that were successful, such as those of Britain, Canada and America.”* (HD 2)

##### 3.2.1.2. Consultations made at the organizational level

The Saudi MoH did not implement and apply strategies from other countries, but they benefitted from the experiences of some companies (national and international) which had extensive experience in this field and a willingness to cooperate with the MoH to create and develop a strategy that was suited to companies in Saudi Arabia. Therefore, consultations were not confined to the country level but were also held at the organizational level. Those organizations played a huge role in the formulation and development of the Saudi e-health strategy. Consequently, the co-operation established a roadmap to facilitate the implementation of EHRs in PHCs in Saudi Arabia; as these comments illustrate:

“*There is no use of outside strategies directly, but we used well-known and large global companies. They have a great experience in this area and they help to set up the private strategy of the Saudi Arabia Ministry of Health, but they did not use other strategies because the e-health strategy of the Ministry of Health was made here in the Ministry, and this strategy won international awards.”* (HD 1)

At organizational level, the “Health Info Way” in Canada was chosen for the establishment and development of the Saudi e-health strategy in general and the implementation of EHRs in particular. At the beginning of the co-operation, the Saudi MoH created a Request for Proposal (RFP) to determine the criteria, requirements, and regulations, as evidenced by the following comments from two participants:

“*We built our Request for Proposal (RFP) and sent it to ‘Health Info Way' in Canada to give it to their consultants; we worked with them.”* (GM 1)

“*We benefitted from ‘Health Info Way' in Canada.” (DHD 1)*

Among the organizations that have been cooperating with them and have had direct involvement in the implementation of EHRs is IBM. The role of IBM is concentrated on the review and implementation of the Saudi e-health strategy and all the projects adopted in the strategy.

“*IBM was responsible for the development of the e-health strategy.” (DA 2)*

##### 3.2.1.3. Consultations made at the individual level

The Saudi MoH also conducted consultations at the individual level. However, this time, the MoH did not rely on international-based experts only, they hired experts from both inside and outside the Kingdom. The task of the internal consultants was to review the decisions made and the strategies; as illustrated in the comments below:

“*Internally we have six reviewers from the Kingdom here.”* (GM 1)

“*…was attended by a number of consultants within the Saudi Ministry.”* (GM 3)

Numerous important issues were discussed with consultants and experts from inside and outside the Kingdom.

“*A lot of reasons for failure are discussed and challenges that may hinder the implementation of the EHRs are also discussed with them (experts).”* (HD 3)

In addition to the internal reviewers, external reviewers from developed countries were hired to review the EHRs implementation plan, and those reviewers had extensive experience in their countries and had participated in successful projects.

“*The Ministry used international auditors and senior experts who have experience in this field.”* (HD 1)

“*It was better to use the same experts who participated in successful strategies in their countries and ask them to participate in reviewing the Saudi E-Health Strategy.”* (HD 1)

The consultants and experts who were involved came from different countries such as Canada, the United States of America (USA), the UK, and Australia, and had extensive experience in this sort of project. The consultations were conducted either by the consultants themselves attending the headquarters of the Ministry or by communicating with them via the Internet; as illustrated by the comments below:

“*We have learned a lot from consultants who have been hired, whether through contracts or being present in the Kingdom, or by communicating with them in their own countries, whether from Canada, Australia or other countries that have experience in these projects. We have been directed by them to do the proper mechanism and how to avoid mistakes.”* (HD 3)

“*We had contact with experts in the field of EHRs implementation from different countries, including USA, Australia and UK.”* (DHD 2)

The selection criteria of the experts and consultants were not limited to their expertise and nationalities. The consultants' and experts' backgrounds, interests, and specialties were also taken into consideration.

“*During the implementation of the system there has been hiring from different backgrounds; different specialties were taken into account. The two parts I had were either from a clinical background or from a technical background.”* (DHD 1)

In addition to the consultations made during the formulation of the plan, the project team also considered the EHRs requirements. The following section illustrates the consideration of EHRs end-user requirements during the planning phase.

#### 3.2.2. EHRs user requirements

The importance of EHRs end-users participation during the planning and EHRs selection phase was illustrated by the participants. The project team at the Saudi MoH pays attention to end-user requirements during planning and software selection.

“*User needs have been considered.”* (HD 1)

“*The end-users' needs and requirements have been taken into account.”* (GM 2)

“*End-users have been taking into account their requirements and needs during the planning.”* (HD 2)

Particularly during the software selection.

“*Essentially, cannot build a system without understanding user requirements.”* (DA1)

“*User needs are the foundation of our system selection criteria.”* (GM3)

#### 3.2.3. Software selection criteria

In addition to the consideration of end-user requirements, this section illustrates other software selection processes and criteria to be considered during software selection. Software selection is one of the major challenges the MoH faces and can lead to delays in many projects, especially the implementation of EHRs in PHCs. According to the head of the department, the Saudi MoH also considers other criteria, such as system efficiency and ease of use of the system, to be important. These two criteria are discussed in detail below.

“*The system's ease of use and efficiency are considered.”* (HD 3)

##### 3.2.3.1. EHRs efficiency

The Saudi MoH considers system efficiency to be one of the requirements of an EHRs implementation project. The participants justified the concerns of the Saudi MoH in this regard since software selection is one of the main reasons for the success or failure of EHRs implementation projects. The deputy head of the department stated that if the efficiency of the system is poor, it will not be able to fulfill its purpose and will not meet the user's expectations, which may lead to user resistance.

“*Efficiency of the system has been taken into account because if the system doesn't help users do their jobs, or if the system doesn't improve health services, user-acceptance will fail, and we may then go back to the paper-based system.”* (DHD 3)

“*This is essential and one of the most important criteria that we have set for the selection of the system -the system's usability and efficiency.”* (GM 3)

##### 3.2.3.2. EHRs usability

The Ministry included ease of use of the system as one of the essential criteria during software selection. To verify the ease of use of any EHRs, the project team is planning to request a trial version of the software from the vendors to test the system. This testing is performed with the coordination of actual users of the EHRs to measure their satisfaction with the system's usability.

“*Ease of use, or so-called ‘user friendliness', is a requirement for each user, and ease of use of the system is part of the criteria that has been set for the selection of any system.”* (DA 1)

“*System usability is one of the most important criteria which we take into account during the testing of any system.”* (GM 3)

##### 3.2.3.3. EHRs interoperability

Interoperability of EHRs systems has also been considered as one of the main criteria during software selection. Participants mentioned two methods of enhancing EHRs interoperability: through the development of such a standard, and the development of terminology scheme. The Saudi MoH is planning to implement interoperable EHRs in PHCs. The purpose of this is to facilitate inter-PHC communication and exchange patient information without any technical problems. This will continue to facilitate the connectivity of PHCs with hospitals and all organizations affiliated with the Saudi MoH, as well as to reduce the huge gap between the regions of the Kingdom of Saudi Arabia.

“*A unified system can be implemented to avoid the problems of compatibility between systems, whether with the same health centers or with hospitals.”* (DA 2)

To enhance systems interoperability, the Saudi MoH is developing its standard.

“*We also started to build our own interoperability standard.” (GM1)*

##### 3.2.3.4. Selecting local or international system

Whether to select a local or an international system is a subject of broad debate among project teams. However, most prefer international systems to local systems, hence the project team primarily endorses the implementation of international EHRs. This is illustrated clearly in the responses of most of the project team.

“*The Ministry has decided to buy an international system.”* (DA 1)

“*The Ministry is looking globally to find a system that meets its requirements. We have to hold many meetings with large international companies that present their systems to the Ministry.”* (HD 4)

However, the head of the department supports a decision to select a local system.

“*One of the things proposed for discussion at the moment is to find a local system for the PHCs.”* (HD3)

##### 3.2.3.5. Prepare RFP

The MoH has formulated an RFP that includes all criteria, requirements, and conditions for the implementation of EHRs. The IT department at the Saudi MoH co-ordinates with the relevant departments to draft the RFP and then provide the companies with a copy. To create an integral and clear RFP, the MoH has conducted a full analysis of the PHCs to determine their functions and requirements. Thereafter, the potential vendors who will implement the EHRs in the PHCs will obtain a copy of the RFP to assess whether they are commensurate with requirements.

“*The IT department team will provide the RFP to companies that have already submitted offers to consider whether their systems meet our requirements.”* (DA 2)

“*A full situation analysis of PHCs has been added to the RFP, which contains all functions and services provided by the PHCs.”* (DA 5)

In the same context, a general manager emphasized the importance of issuing a clear and integral RFP document to avoid any problems during the implementation of EHRs.

“*It is very risky if the RFP is not clear for the vendor; you may face implementation issues in the future.”* (GM1)

In addition to software selection, training and awareness campaigns were discussed during the formulation of the plan. The following section will describe the plan to introduce the system through awareness campaigns.

#### 3.2.4. Training provision

As mentioned earlier, training will be provided side by side with the awareness campaigns. Therefore, the Saudi MoH has paid attention to the provision of training, setting up several training courses, and developing a proper plan to provide training for all EHRs users.

“*We are prepared to arrange suitable training programmes for users.”* (DA 1)

“*Training is very important to ensure the success of the EHRs usage on an ongoing basis.”* (HD 1)

Moreover, the majority of participants agreed that training courses are essential to the EHRs implementation plan. For example:

“*The training courses received much attention in the planning.”* (HD 1)

“*Training is a key element in the planning.” (DHD 1)*

On the other hand, interviewees illustrated that training courses will be presented in two languages (Arabic and English) to be accessible to all end-users, even non-Saudis.

“*The content of training materials will be printed in two languages, English and Arabic, for the convenience for all users.” (GM1)*

“*Training courses will be presented in both Arabic and English languages.” (DHD 2)*

##### 3.2.4.1. Training delivery methods

Training courses will be implemented in a number of ways, including requesting the health affairs administration in each region to hold training courses for the EHRs users in that region. In addition, the MoH will distribute guide leaflets to help EHRs end-users to understand the system.

“*Training will be centralized through directing the mission to the regions' administrations, where each health affairs management ensures to train their PHC staff.”* (HD 3)

“*With the distribution of guided leaflets to make it easier for the user to use the system, they can educate themselves without the need for trainers.”* (DA 1)

Another planned method by the Saudi MoH to provide training to all EHRs end-users is the concept of trainer training. One member from each PHC will be trained and then this member provides training to his/her colleagues at the same PHCs.

“*We will train one employee who, in turn, will train another employee in the PHC.”* (DHD 1)

“*Train some users; after that the users will train new employees in the future.”* (DA 1)

##### 3.2.4.2. Training provision as a condition of the contract with vendors

The Saudi MoH is planning to include a clause in the contract with selected vendors to take the role of providing training courses for EHRs end-users.

“*One of the contract terms put to the vendor is to train the users.”* (HD 1)

“*Other options would be for the vendors to arrange and provide training courses for users.”* (DHD 2)

##### 3.2.4.3. Training provision timeline

As planned, the training sessions will be held in the early stages of the implementation of EHRs (pre-implementation phase).

“*The training courses will be held in the pre-implementation stage.”* (GM 2)

“*At the beginning of the project, training courses will be provided.” (HD 1)*

Others stated that the training begins in the early stages of an EHRs implementation project and will last throughout all project phases, including the post-implementation phase.

“*Training will be on an ongoing basis from the beginning of the implementation; even after the implementation.”* (GM 3)

“*This will be ongoing during the implementation and after.”* (HD 2)

#### 3.2.5. Technical support provision

In addition to training, technical support is another element discussed during the planning of EHRs implementation in PHCs. Technical support is one of the pillars of the project, and it is provided on an ongoing basis after the implementation of EHRs, especially in the post-implementation phase. In addition, the participants emphasized that technical support is provided from within SA and not from outside the country if the EHRs are implemented by an international vendor.

“*Technical support is very important, and it is important to have technical support from within SA and not from the country we are buying the system from.”* (HD 1)

In the same context, technical support will be provided remotely through call centers in the headquarters of the MoH. However, this method is subject to the connectivity between the MoH and the PHCs.

“*There is a huge call center in the MoH to provide technical support. We are hoping to get the connectivity to provide remote support to the PHCs.”* (DA 3)

##### 3.2.5.1. Provision of technical support as a condition of the contract with vendors

As planned, similar to training, technical support will be provided by the vendors who implement the EHRs. Therefore, the Saudi MoH obliges the vendors to take responsibility for providing technical support on an ongoing basis, which will be included as a clause in the contract with the selected vendors.

“*We will ask the companies to provide technical support constantly by adding some items to the contracts before signing.”* (HD 3)

“*Technical support will be considered and approved during the agreement of any contract with any vendor.”* (HD 2)

In this context, technical support will be conducted on three levels, as follows: MoH level, represented by the call center, service provider level, represented by the Telecom Communication Companies (TCCs), and vendor level.

“*Usually, there will be three levels of technical support: (1) through the call centers at the Ministry; (2) services providers such as Saudi telecom companies; (3) through the vendor.”* (HD 3)

#### 3.2.6. Security, privacy, and confidentiality assurance

During the planning phase, the project team considers issues related to the protection of patient data by taking several steps to improve security system standards to maintain patient confidentiality. Such steps were taken at both technical and human levels. Moreover, the Saudi MoH applied very strict rules and regulations to prevent any irregularities or breaches of patient data. The main procedures conducted by the MoH are monitoring, implementing data protection law, applying non-disclosure agreements, giving privileges to users, and selecting secure systems.

“*There is a big focus on security and protection of patient data as well as data confidentiality and privacy.”* (DA 1)

“*There are policies that have been developed for security and confidentiality.”* (DA 3)

##### 3.2.6.1. Data protection laws to protect patient data

The Saudi MoH has adopted laws to protect patient data from unauthorized access and any attempt to use it illegally. These laws involve deterrent punishments for any unethical use of such information.

“*There are also systems and governmental laws concerned with punishing and preventing any illegal usage.”* (GM 1)

“*Strict standards and penalties for any use not in a moral position.”* (DHD 1)

Moreover, the MoH prompted all EHRs users to sign a “non-disclosure agreement” which contains clauses that ensure the protection of patient information from any misuse. In addition, the MoH sends a “confidentiality letter” to all EHRs users and vendors. Through this document, the user undertakes not to disclose any information concerning the patient or to use patient data for any purpose other than healthcare.

“*The confidentiality of data is very important and, based on this matter, the Ministry in turn has prompted all its employees to sign a Non-Disclosure Agreement.”* (HD 1)

“*A ratification has been made and should be signed by the vendors and the users themselves. It is called a Confidentiality Letter; the ratification contains a clause stating not to use any information for any other purposes.”* (HD 3)

##### 3.2.6.2. Monitoring and auditing by PHC directors

The Saudi MH is planning to monitor these systems by granting PHC directors the authority to monitor all transactions made by PHC staff to detect any misuse during the use of these systems.

“*The Ministry not only endorsed this ratification, but carries out audits and follow-ups of all electronic transactions. In the case of proven misuse, there is a penalty.”* (HD 3)

“*We have given each center director the power to monitor, so the director knows exactly what each employee does, how many orders have been entered and how many patients have been served.”* (SD 1)

##### 3.2.6.3. Limited access privileges

All EHRs end-users from different levels and occupations will be provided limited access based on their role. Therefore, privileges will be granted to those users to avoid any unauthorized access.

“*…privileges will be determined for each user.”* (HD 1)

“*In terms of users themselves, they will be given privileges based on each user's role and responsibilities.”* (GM 1)

##### 3.2.6.4. Selecting a secure system

In addition to the procedures mentioned above, system security will be taken into consideration during software selection.

“*One of the Ministry's criteria for the selection of any EHRs is that the system should be secure and not penetrable. We will test any system before we purchase it.”* (HD 3)

### 3.3. Theme three: procedures adopted in the pre-implementation phase

This theme will describe EHRs pre-implementation phase procedures for PHCs in SA. It will be presented as a process based on the codes generated from the transcripts. The pre-implementation procedures began with selecting the project team. Once the project team selection has been described, the participants explained the communication mechanisms between the project team. In addition, the participants explained how end-users and other stakeholders were involved. Thereafter, they underline how to maintain EHRs end-user acceptance. The interviewees also illustrated the process of readiness assessment, PHCs restructuring, and workflow redesign.

#### 3.3.1. Project team selection

The individuals on the project team were chosen carefully by the IT department at the Saudi MoH in accordance with their ability to carry out this task.

“*In the implementation, well-qualified people were selected.”* (HD 3)

“*All the team members have been selected by the IT department and they are all highly qualified.”* (HD 2)

The project team consists of three different levels: senior managers at the Saudi MoH level and health affairs management at the regional and PHC level.

“*The selection of project team members was from three levels: senior management at the Ministry, and then from the regions' management level, and finally, from the PHC level.”* (DAs 2)

According to the software developer and one of the data analysts, the project team consisted of doctors, nurses, technicians, laboratory IT technicians, engineers, and administrators. In addition, the project team has experience in their field and others have experience in similar projects.

“*This is natural, they were appointed as highly qualified people from different departments and expertise; so, we selected doctors and engineers, including a doctor who worked for 10 years as a PHC manager and served us in the analysis of the current status of PHCs.”* (SD 1)

“*The team was composed of several different levels and backgrounds of doctors, nurses, administrators, lab technicians and center managers.”* (DA 1)

Similarly, HD3 stated that the project team consists of people “*from various departments and all levels.”*

Once the project team had been selected, the project team members started communicating among themselves. The following section will describe the project team's communication mechanisms.

#### 3.3.2. Project team communication

The Saudi MoH has utilized methods to ensure proper communication among the project team members. Communication between the project team members is conducted through the formation of committees and the holding of workshops and meetings, and occurs either face-to-face at the headquarters of the MoH or via the Internet and other media.

##### 3.3.2.1. Communications are made via committee

The formation of committees is one of the most common methods used by the Saudi MoH to conduct consultations and negotiations as well as to make decisions related to EHRs implementation in PHCs in SA. Committees often consist of representatives from the relevant departments and the PHCs. In addition to direct encounters in person at the Ministry, members of the committees communicate either through e-mail or mobile phones.

“*Committees have been formed to communicate with other project team members.”* (HD 3)

“*…and set up specialized committees for this project with the involvement of representatives from different departments at the headquarters of the MoH.”* (GM 3)

Communication among all project team members is not limited to committees, and workshops are also used to communicate between project team members.

##### 3.3.2.2. Communications were made via workshops

Project team communication workshops are held inside the headquarters of the MoH to promote proper communication between project team members. The workshops are held on an ongoing basis.

“*We hold continuous workshops.” (GM 2)*

“*One of the most important plans made by the Ministry to ensure good communication between the project team is holding repeated workshops and periodic meetings; often weekly.”* (DA 1)

Some workshops are even held outside work hours, which is reportedly the most effective method of communication for project team members.

“*We hold the communication process and meetings and workshops outside of work hours.”* (GM 3)

##### 3.3.2.3. Communications made via meetings

In addition to the formation of committees and the holding of workshops, the project team holds regular meetings to ensure communication among all members of the project team.

“*Communication between teamwork through holding regular meetings.” (HD 2)*

“*We made regular meetings.”* (SD 1)

##### 3.3.2.4. Communications made via media

The typical method of communication between the project team is email, video conferencing, and other media.

“*We communicate through email as well as video conferences to facilitate communication between us.”* (DHD1)

“*Communicate via e-mail as well as through mobile.”* (HD3)

The interviewees also highlighted the importance of involving EHRs end-users and other stakeholders in the above-mentioned communication methods. The following section will describe the EHRs end-users' and other stakeholders' mechanisms.

#### 3.3.3. EHRs end-user involvement mechanisms

End-user involvement leads to enhance EHRs end-user satisfaction. Hence, EHRs end-users have been involved through committee-appointed representatives. These representatives can be champions or super-users who act as a communication channel between the project team and the end-users.

“*Representatives from the PHCs have been appointed for the representation of other users.”* (HD3)

“*The goal of involvement is to reduce the rate of resistance and unwillingness to use the system.”* (GM3)

The involvement of EHRs end-users took place in the early stages of the EHRs implementation project. Thus, planning was formulated with consideration of the involvement of the stakeholders. However, end-user involvement was not limited to the early stages, but lasted throughout all phases of the project, even into the post-implementation phase.

“*All beneficiaries of the system or stakeholders have been involved since planning and strategy development.”* (HD1)

“*We have involved all stakeholders in the strategy; they were always participants in our meetings, and we are still carrying out these meetings and consultations between us.”* (GM3)

On the other hand, the level of participation in the project varied from one phase to another. There was reasonable involvement in the process of decision-making in general. The MoH did not make any decisions concerning the implementation of EHRs without consulting all those involved; each according to his/her specialization, particularly in clinical decisions. Inputs were taken from representatives of the PHCs from different backgrounds.

“*All stakeholders were involved in decision-making.”* (DA 3)

“*There is no decision made with respect to clinical matters without consultation and participation of a doctor or other technician who specializes in the same field.”* (HD 1)

In this context, the EHRs end-users or their representatives were particularly involved in the decision-making regarding software selection.

“*Stakeholders were involved during the software selection.”* (*HD 1)*

“*Whenever we want to select a new system, we should engage stakeholders or their representative.”* (GM 1)

There is a correlation between EHRs end-user involvement and their acceptance of EHRs implementation. Therefore, the interviewees underline the importance of maintaining end-user acceptance in the early stages. The following section will describe the procedures followed to maintain end-user acceptance of EHRs implementation.

#### 3.3.4. User acceptance of new EHRs implementation

Human-related factors were a focus of the project team during EHRs implementation.

“*…in particular human factors; they are the most important factors that may contribute to the success or failure of EHRs implementation.”* (HD3)

For instance, EHRs end-user acceptance and satisfaction were found to be crucial to the success of the project.

“*If the user doesn't want to use the system, then it will affect the success of the implementation.”* (DA 1)

Therefore, the Saudi MoH now attempts to prepare EHRs end-users for the changes.

“*Preparing them and trying to remove the fears of using the new technology.”* (HD 1)

The responses obtained from some of the participants indicated that the majority of users are very enthusiastic about the use of EHRs and are motivated to make the transition to electronic transactions in their workplace.

“*They are ready for the maximum extent because they need this system; electronic transactions are required to facilitate the performance of work.”* (HD 3)

“*Users are ready, and they are very enthusiastic about the system.”* (DA 1)

One of the encouragements that contributed to the EHRs end-user's enthusiasm and acceptance was the positive impact of technology on their daily lives.

“*Most users were briefed on the technical side of things and they know the value of this technology and its impact on their daily lives.”* (GM 3)

#### 3.3.5. Awareness campaigns to EHRs users

To prepare the end-users for the new EHRs and reduce their resistance to it, awareness campaigns will be carried out and occur side by side with training in the implementation process. The awareness campaigns will be presented continuously throughout all implementation phases.

“*There will be awareness campaigns and guidance during the implementation period.”* (HD3)

In this context, the delivery methods used for awareness campaigns will be through distributing brochures and leaflets explaining the EHRs project as well as the intended benefits of the system. In addition, there will be visits to the PHCs to introduce the implementation of the EHRs project.

“*At the beginning, there will be the provision of promotional material such as leaflets.”* (HD 1)

“*…brochures will be distributed prior to the implementation and visits to these PHCs will be held to introduce the EHRs.”* (HD2)

However, a general manager argued that the Saudi MoH “*In marketing; is not strong enough.”* (GM1)

#### 3.3.6. Readiness assessments of new EHRs

Healthcare organization's readiness to implement new EHRs is influential to the success of the project. Therefore, the Saudi MoH takes several readiness measurements to ensure successful EHRs implementation.

“*Some of the requirements have been considered, such as the readiness of PHCs to successfully implement new technology.”* (GM 1)

Numerous studies were conducted to identify all the obstacles and facilitators directly associated with the success or failure of EHRs implementation projects. In addition, the Saudi MoH conducted research to determine the strengths and weaknesses that may influence EHRs implementation in PHCs in SA.

“*Firstly, we made so many studies prior to the implementation to know the strengths and weaknesses of the challenge.”* (DHD 1)

“*We conducted a number of studies and research before the beginning of any EHRs implementation to learn the strengths and weaknesses, as well as to determine the challenges, the causes of failure and identify risks.”* (HD 3)

At a technological level, the MoH is currently assessing the readiness of PHCs, in regard to the connectivity infrastructure, to accommodate the EHRs implementation project and identify any obstacles and challenges.

“*We discussed the readiness of hospitals and PHCs in terms of infrastructure compatibility with the change, especially PHCs.”* (HD 3)

“*We are currently making a situation analysis of the PHCs, including an assessment of the PHCs readiness in terms of connectivity.”* (GM 3)

While some of the participants believe all PHCs are currently ready for EHRs implementation,

“*PHCs are currently ready to implement the EHRs.”* (DHD3)

Others think that PHCs are not yet ready to implement the EHRs.

“*I think some of the PHCs are still not ready for this project, compared with hospitals.”* (SD 1)

“*Some of the PHCs need to be prepared for the new implementation - they are not ready yet.”* (GM1)

#### 3.3.7. Restructuring and workflow redesign of PHCs to new EHRs

In the pre-implementation phase, the Saudi MoH is planning to re-structure the workflow of the PHCs to comply with new changes post EHRs implementation. This is confirmation that the MoH is restructuring the workflow of some of its centers that use EHRs. Although there will be three different vendors implementing EHRs in Saudi PHCs, they will take the role of redesigning a unified workflow. According to one of the participants, the workflow redesign will not be affected by the fact that the Ministry will select three different vendors.

“*If you go to the PHCs that have the EHRs and other centers, you will see that they have been redesigned to comply with new IT projects.”* (GM1)

## 4. Discussion

Initially, the project team members agreed that “*there is a lot of knowledge about the on-going development needs of EHRs support staff”*. However, it is worth noting that the project at the Saudi MoH relies on capitalizing on the experiences of other organizations and other countries in the development of strategic implementation plans and policies. The study illustrated a moderate level of readiness in terms of training and technical support. It was indicated that awareness campaigns are essential and can be included as part of training courses. Similar to this study's findings, Steininger et al. (38), and Allen et al. (39) also found that providing adequate awareness campaigns would contribute to enhancing end-user readiness for the introduction of new EHRs and reduce their resistance. Therefore, it is highly recommended to provide awareness campaigns for large-scale projects that may face direct contact challenges with stakeholders. According to the Saudi MoH plan, training will be provided to EHRs end-users before implementing a system to raise their competence level and avoid any resistance or training issues.

Moreover, the planning has taken into consideration several methods to provide adequate training and support. The study findings and those of Piliouras et al. (40) and Keshavjee et al. (41) determined that relying on vendors to provide training and support to end-users is a useful way to overcome any challenges related to the provision of training and support, particularly in large-scale projects. In addition, the approaches planned by the MoH involve training a large number of staff for a large-scale implementation project so these trainers can then train the end-users; this concept is known as “train the trainers”. These findings are consistent with data obtained from previous studies (42) which suggest applying the concept of “train the trainers”. It is of note that the study by Slight et al. (42) was conducted in secondary care in the UK. Other possible training methods have been agreed upon, such as distributing guidance leaflets to help end-users and educate them.

As identified in this study, project team communications were made through three different methods: meetings, committees, and workshops. Regular meetings and workshops were also found to be useful methods of ensuring proper communication among project team members in previous studies conducted in developed countries (43, 44).

As part of the project team's preparation for the implementation of this large-scale project, they conducted several consultations to enhance the readiness of the PHCs as well as to formulate a well-defined plan. As revealed in this study, consultations were found to have a prominent role in enhancing the success of the implementation of this large-scale project as well as overcoming any barriers that may have hindered its success. One of the most interesting findings to emerge from the semi-structured interviews is that such a large-scale project should consider several consultations and attempt to benefit from the experiences of other countries or experts with extensive experience in the field of EHRs implementation. This is due to the magnitude of the project and the need to take extreme care to avoid mistakes that can be extremely costly. Reliance on consulting services and taking advantage of the experience of others are positive measures that will contribute to the success of any implementation project. The findings from this study suggest that conducting a multi-stage consultation during the planning phase could avoid mistakes or drawbacks that may lead to project failure. These are country level, organization level, and individual level. Conducting consultations can assist to overcome the shortage of HI experts.

The findings from this study identify that the business structure of PHCs has been redesigned to comply with EHRs implementation, which indicates that PHCs are ready for the EHRs at the process level. In contrast, healthcare organizations in the UK have recorded lower readiness due to the variation between workflow processes and new IT systems (6). Similarly, the findings illustrated that PHCs are at a high level of readiness at the management structure and administrative and financial support level. However, due to a lack of awareness of the PHC staff with regard to certain administrative aspects related to EHRs implementation in Saudi PHCs, findings about management structure were mostly obtained from the project team.

A well-designed strategic plan can directly link with consultations made during the planning phase, especially for large-scale projects. In contrast, the readiness of healthcare organizations in the USA was found to be low at the planning level (45). On the other hand, the study findings illustrated that PHCs are at a lower level of readiness with respect to technology than they are in relation to other factors such as resources. Infrastructure aspects such as connectivity were recorded to be inadequate and not meeting the requirements for EHRs implementation. These issues represent a challenge for the Saudi MoH. These findings are in line with Lennon et al. (6), and Sahay (46) who also argued that developing countries are still behind developed countries in terms of technical infrastructure. However, Cherry (45) reported a low level of readiness in developed countries such as the USA with respect to the provision of appropriate hardware. Lennon et al. (6) also found that the level of readiness in both hospitals and PHCs in the UK was low with respect to technical infrastructure. Furthermore, Biruk et al. (47) documented low infrastructure readiness in Ethiopian hospitals. On the contrary, Saleh et al. (48) found that PHCs in Lebanon were at a higher level of readiness in terms of hardware. Most importantly, large-scale projects can suffer dramatically due to poor infrastructure, systems interoperability, and other technological challenges. If these challenges are insurmountable and improperly handled, they may lead to the failure of EHRs implementation projects. All of these findings show that poor technical infrastructure was the most significant factor affecting readiness for EHRs implementation in healthcare organization in developing and developed countries alike, especially when implementing large-scale projects.

The findings show that EHRs end-users gave positive feedback about data accessibility, accuracy, improved productivity, and time-saving as a result of the system. Although these findings differ from those of several published studies (49–54), which argue that EHRs decrease staff productivity, they are consistent with those of Cheriff et al. (55) and Lorenzi and Kouroubali (56). On the other hand, the findings revealed resistance toward EHRs implementation. Dissatisfaction with the implementation of the EHRs also documented among end-users in SA. Sixty-one percent of the participants claimed that they had abandoned the new EHRs and had gone back to the historical paper-based system (57). In contrast, in Arab Gulf Countries (AGCs), the EHRs end-users have recorded a high level of satisfaction with the implementation and use of EHRs in secondary care (58, 59). Variable satisfaction toward the implementation of EHRs was also recorded in non-AGCs. While high levels of EHRs end-user's satisfaction have been recorded in several studies (60–62), others have shown that EHRs end-users express moderate levels of satisfaction (63–65). In contrast, low levels of satisfaction or dissatisfaction have been expressed by EHRs end-users toward the use and adoption of the system in other studies [e.g., (66–68)].

### 4.1. Study contributions

- This study provides lesson learned experience to implement large-scale EHRs in the PHCs which include procedures, processes, and recommendations.
- This study also described the pre- and post-implementation of EHRs which can be used as a guideline for project managers and policymakers.
- This study generated several factors that influence the EHRs implementation which can be quantitively examined.
- The findings of this study are based on two different projects in the same context.

## 5. Conclusion

This study explores EHRs implementation in Saudi PHCs from a project team perspective. The majority of the data addresses the pre-implementation and post-implementation phases. This provides two different experiences and lessons learned from unsuccessful attempts. Initially, the findings reveal that the majority of factors influencing EHRs implementation were taken into consideration by the project team during the pre-implementation phase. For instance, organizational level factors such as training, support, legal issues, and organizational workflow and redesign were a concern of the project team during the pre-implementation phase. In addition, other factors related to technology and end-users were included in the EHRs implementation plan.

The evaluation revealed that it was implemented in 150 PHCs and was considered as a pilot to the previous project. The evaluation also revealed that the main causes that lead to the failure of the previous project were lack of connectivity, lack of technical support, and staff changes, particularly those who occupied high-level positions in the Saudi MoH. However, the evaluation also revealed that the implemented EHRs were easy to use, efficient, and improved healthcare quality and end-user productivity.

Conducting consultations was found to be a predictor of the level of readiness of the healthcare organizations for the introduction of large-scale EHRs, and only emerged following the analysis of this study. Therefore, future researchers who are interested in assessing healthcare organizations' readiness to implement EHRs, especially large-scale projects, may need to consider this issue and its impact on the project's success.

## Data availability statement

The raw data supporting the conclusions of this article will be made available by the authors, without undue reservation.

## Ethics statement

The studies involving human participants were reviewed and approved by the Institutional Review Board (IRB) of King Fahad Medical City (KFMC) at the Saudi MoH (IRB Log No. 14-189E). The patients/participants provided their written informed consent to participate in this study.

## Author contributions

HA: conceptualization, methodology, data collection, validation, analysis, and writing.
